# Laparoscopic gastrectomy reduces adverse postoperative outcomes and decreases morbidity for gastric cancer patients with visceral obesity: a propensity score-matched analysis

**DOI:** 10.7150/jca.47552

**Published:** 2021-02-21

**Authors:** Chenchen Mao, Xiaodong Chen, Xiangwei Sun, Xiang Wang, Ce Zhu, Wenjing Chen, Xiangyang Xue, Xian Shen

**Affiliations:** 1 Department of Gastrointestinal Surgery, The Second Affiliated Hospital, Wenzhou Medical University, Wenzhou, Zhejiang, China.; 2 Department of Gastrointestinal Surgery, The First Affiliated Hospital, Wenzhou Medical University, Wenzhou, Zhejiang, China.; 3 Department of Medical Microbiology and Immunology, Basic Medical College, Wenzhou Medical University, Wenzhou, Zhejiang, China.

**Keywords:** obesity, abdominal, laparoscopy, stomach neoplasms, postoperative complications, survival

## Abstract

**Background:** Laparoscopic gastrectomy for gastric cancer shortens the recovery period without decreasing long-term survival. However, clinical evidence on whether laparoscopic radical gastrectomy reduces the surgical stress and improves the short- and long-term outcomes of obese patients with gastric cancer is lacking. We compared the short- and long-term outcomes of gastric cancer patients with visceral obesity (VO) who underwent laparoscopic gastrectomy (LG) or open gastrectomy (OG).

**Methods:** We prospectively collected data from 578 patients who underwent radical gastrectomy in two centers between January 2014 and December 2016. The visceral fat area (VFA) was measured on the umbilicus level, and VFA ≥100 cm^2^ was defined as VO. The section bias was reduced by conducting a propensity score matching analysis. The short- and long-term outcomes were further compared between patients who underwent OG and those who underwent LG.

**Results:** Overall, 245 patients (42.61%) were classified as having VO, of whom 102 were included for further analysis after matching. There were no significant differences in clinical characteristics between the two groups in the matched cohort. The LG group had significantly fewer overall complications (P<0.001) and shorter postoperative hospital stays (P<0.001). Subgroup analysis of postoperative complications also showed that the incidence of surgical complications was lower in the LG group (P=0.002). Further survival analysis showed the LG group had significantly better long-term overall survival (P=0.017).

**Conclusions:** Compared with open radical gastrectomy, laparoscopy would reduce the rate of postoperative complications in patients with VO, as well as prolong their overall survival.

## Introduction

Gastric cancer (GC) is the fourth most common cancer worldwide and the third leading cause of cancer-related mortality, with 723,000 deaths annually 1, 2. Obesity has been reported to be associated with poor surgical outcomes in GC, including fewer lymph node dissections and more postoperative complications 3, 4. Traditionally, body mass index (BMI) has been broadly used to indicate obesity 5, 6. However, BMI cannot distinguish fat distribution within the intra-abdominal cavity or different types of adipose tissue 7. Several studies recently proposed that visceral fat is likely to be a more optimal tool for predicting surgical outcomes, and visceral obesity (VO) is superior to BMI for the prediction of complications after colonic and gastric surgery 8-11. Therefore, studies should examine the amount of body fat and use VO in clinical practice and research.

Laparoscopy-assisted gastrectomy (LG) has been rapidly adopted for GC 12. Multiple studies have shown that compared to traditional open surgery, laparoscopic radical gastrectomy has the apparent advantage of minimizing invasive procedures and shortening the recovery period; laparoscopy also results in comparable long-term survival 13-15. However, the thicker abdominal wall in obese individuals increases the operation difficulty and postoperative infection risk. Studies have also shown that obesity may be a contraindication for laparoscopic surgery 4. There is still a lack of clinical evidence whether laparoscopic radical gastrectomy can reduce the surgical stress and improve the short- and long-term outcomes of obese patients with GC.

The purpose of our study was to provide a reference for the clinical implementation of the best surgical approach by comparing and analyzing the short- and long-term outcomes of GC patients with VO who underwent different surgical procedures.

## Materials and methods

### Study design and patient population

Data were prospectively collected from patients who underwent R0 gastrectomy and D2 lymphadenectomy at the Gastrointestinal Surgical Departments of the Second Affiliated Hospital of Wenzhou Medical University and the First Affiliated Hospital of Wenzhou Medical University in China between January 2014 and December 2016. The choice of open or laparoscopic surgery was based on the doctor's advice and patient's decision after reading the informed consent form. This study was approved by the ethics committees of the Second Affiliated Hospital of Wenzhou Medical University, and all participants provided written informed consent prior to study participation.

### Inclusion/exclusion criteria

All adult patients who met the following criteria were included in the analysis: (a) patients with histopathologically confirmed gastric adenocarcinoma and scheduled to undergo radical gastrectomy; (b) patients aged ≥ 18 years; and (c) patients who provided written informed consent to participate in the study. The following exclusion criteria were applied: (a) patients who lacked imaging data; (b) patients who underwent palliative surgery or emergency surgery; (c) patients who received neoadjuvant chemotherapy or radiotherapy; (d) patients who had a severe immune, blood, or endocrine disease; (e) patients with GC concurrent with other malignant tumors; and (f) patients with substantial absence of clinical data. Operations were performed per the Japanese Gastric Cancer Treatment Guidelines 2010, version 3.

### Baseline data collection

All data were collected prospectively and maintained in a digital database. For each patient enrolled in this study, demographic details, including age, sex, BMI, American Society of Anesthesiologists (ASA) grade, abdominal operation history, and NRS 2002 score were collated. Other details pertaining to the operation, such as tumor location, tumor differentiation, pathological classification, and histopathologic staging according to the TNM staging (AJCC Cancer Staging System, 8^th^ ed), were also collected. Additionally, postoperative outcomes according to the Clavien-Dindo classification 16, postoperative hospital stays, hospitalization costs, and overall survival (OS) data were also collected.

### Computed tomography-based measurement of visceral fat area

Preoperatively, all patients underwent computed tomography of the general abdominal cavity. We selected a single cross-section scan at the umbilicus level for quantification of the degree of visceral fat. A threshold of -140 to -50 was used for visceral fat, comparable to the methods in previous studies 17-19. The total fat area was calculated using a dedicated processing system (version 3.0.11.3, BN17 32-bit; INFINITT Healthcare Co., Ltd., Seoul, South Korea). Patients with a visceral fat area (VFA) larger than 100 cm^2^ were classified as having VO, as suggested in the previous literature 6, 11, 20.

### Follow-up

Trained doctors were responsible for visiting the patients and conducting a phone call to follow up with the patients after surgery. The last follow-up evaluation was conducted in January 2019. Overall survival (OS) was defined from the day of surgery until death or until the final follow-up date in January 2019, whichever was first.

### Statistical analyses

To compare the laparoscopic group and the open group, propensity scores were generated using a logistic regression model on the all the baseline covariates: age, BMI, NRS score, ASA grade, hypertension history, diabetes mellitus history, abdominal surgery history, tumor location, TNM stage, differentiated degree, pathological type, and combined resection. Propensity score matching (PSM) was performed in a 1:1 ratio, and an optimal matching with a caliper size of 0.03 was used to avoid poor matches. The two matched groups were evaluated with respect to the study endpoints. Means and standard deviations were used for all continuous data, and numbers and percentages were calculated for all categorical data. In univariate analyses, the independent t-test and Mann-Whitney U-test were used to analyze intergroup differences in continuous variables. The chi-square test and Fisher's exact test were applied to categorical variables. In multivariate analyses, conditional logistic regression analyses were performed to evaluate the association between patient characteristics and short-term outcomes. OS was defined as the time between the date of diagnosis and the date of death or last known follow-up. The Kaplan-Meier method and log-rank test were used to estimate and compare survival, respectively, based on specific factors. The Cox proportional hazard model was used to estimate the risk ratio in univariate and multivariate analyses, and results were expressed as odds ratios (ORs) with 95% confidence intervals (CIs). All P values were two-sided, and P < 0.05 was considered statistically significant. All statistical analyses were performed using IBM SPSS Statistics for Windows/Macintosh, version 22.0 (IBM Corp., Armonk, N.Y., USA) and R version 3.0.1 (https://www.r-project.org).

## Results

### Patient characteristics

**Figure 1:**
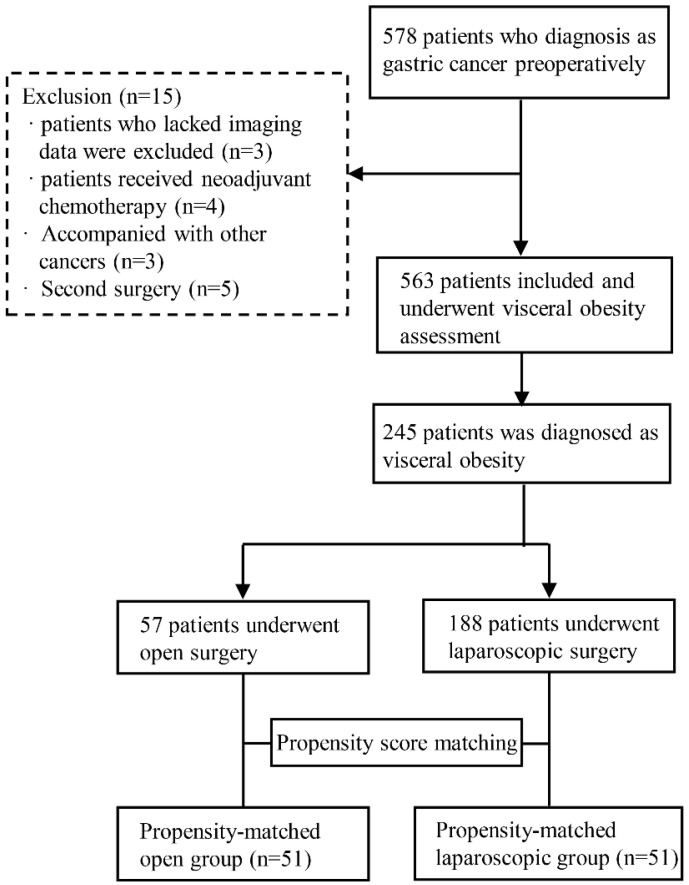
Flow chart of the study procedure.

Among the initially recruited 578 patients, 15 were subsequently excluded, and the reasons for exclusion are detailed in Figure 1. Of the 563 remaining patients, 245 had VO and were therefore included in the study.

As summarized in Table 1, laparoscopic surgery and open surgery were performed in 57 patients (23.27%) and 188 patients (76.73%), respectively. LG was more likely performed in patients with lower TNM stage (P=0.006). Further, patients who underwent laparoscopic surgery were significantly younger (P=0.003) and had lower NRS scores (P=0.039) and ASA stage (P=0.013) than patients who underwent open surgery. There was no significant difference in sex, BMI, the incidence of hypertension or diabetes mellitus, abdominal surgery history, tumor location, tumor differentiation, pathological classification, or combined organ resection between the two groups. After PSM, the total cohort comprised 102 patients: 51 patients comprised the laparoscopic gastrectomy (LG) group and 51 patients comprised the open gastrectomy (OG) group. As shown in Table 1, the two groups of patients were well matched, and there were no significant differences between the groups after PSM.

### Laparoscopic surgery was an independent protective factor against postoperative complications

In the matched cohort, 28 of 102 patients (27.45%) experienced postoperative complications (Table 2). The postoperative complication rate was significantly lower in the LG group than in the OG group (11.76% versus 43.14%, P<0.001). Further analysis of complications between the two groups showed that the rate of surgical complications was significantly lower in the LG group than in the OG group (5.88% versus 29.41%, P=0.002). Other complications were also lower in the LG group, but no statistical significance was found. Additionally, no patients suffered 30-day mortality, and only one had a second operation after open gastrectomy. There were no significant differences between the two groups of patients in the type of surgery. However, more patients underwent Billroth-I reconstruction in the laparoscopic group than in the open group (Table 2).

Univariate and multivariate analyses of factors associated with overall postoperative complications are summarized in Table 3. In the univariate analysis, overall postoperative complications were significantly associated with laparoscopic surgery (P=0.001). Further multivariate logistic regression analysis also showed that laparoscopic surgery was an independent protective factor for postoperative complications (OR 0.141, 95% CI 0.042-0.472, P=0.001).

### Laparoscopic surgery was independently associated with better OS

The mean follow-up was 33.92 (± 11.32) months. There was no significant difference in the follow-up period between the LG and OG groups (35.36±8.35 versus 32.47±13.60 months, P=0.200). As shown in Figure 1, patients in the LG group had a better outcome than that in the OG group (P=0.017).

Further evaluation of the potential factors influencing OS was performed. A univariate analysis showed that OS was affected by a higher NRS score (NRS 3-4: HR 1.698, 95% CI 0.540-5.341, P=0.366; NRS 5-6: HR 6.180, 95% CI 1.361-28.055, P=0.018), TNM stage (TNM II: 4.758, 95% CI 0.871-25.995, P=0.072, TNM III: 10.492, 95% CI 2.321-47.434, P=0.002), and the type of surgery (Laparoscopy: HR 0.280, 95% CI 0.091-0.859, P=0.026). A multivariate analysis showed that a higher NRS score (NRS 3-4: HR 2.588, 95% CI 0.579-11.570, P=0.213; NRS 5-6: HR 12.968, 95% CI 1.780-94.495, P=0.011) and TNM stage (TNM II: 5.686, 95% CI 0.737-43.861, P=0.095, TNM III: 17.492, 95% CI 2.445-125.157, P=0.004) were independently associated with worse OS, whereas laparoscopic surgery (HR 0.264, 95% CI 0.077-0.905, P=0.034) was an independent protective factor (Table 4).

## Discussion

In the current study, we first tried to specifically compare the effect of LG and OG on the surgical outcomes in GC patients with VO. Interestingly, compared with open radical gastrectomy, laparoscopy gastrectomy would reduce the rate of postoperative complications in patients with VO and prolong their OS.

According to a recent study, the number of overweight or obese people has already surpassed the number of underweight people. Notably, China has the largest number of overweight people (more than 89.6 million in 2014) 21. Problems related to obesity have attracted an increasing amount of attention from surgical specialists since a large amount of adipose tissue in the abdominal wall and the abdominal cavity greatly increases the difficulty of exposing the operative field in laparotomy 4. Studies also indicate that, compared with non-obese patients, obese patients experience significantly longer operation times, more intraoperative blood loss, and are more likely to develop postoperative complications after abdominal operation 4, 20.

Considering the priority of VO over BMI in several studies 11, 20, we used CT-based-VFA for determining VO in this study and observed a high incidence of VO in patients with GC (42.61%). Therefore, it is necessary to focus on the clinical effect of VO in patients with GC. Additionally, although several studies 22, 23 have already shown that LG is superior to OG in short-term outcomes, no such studies focus on whether the minimal invasiveness of laparoscopic GC surgery can reduce the surgical stress on obese patients, improve their tolerance to surgery, or improve their short- and long-term outcomes.

In the present study, patients in the LG group experienced fewer postoperative complications and shorter postoperative hospital stays than patients in the OG group. Additionally, LG turned out to be the only independent protective factor for total postoperative complications, which was similar to the results of a recent study which demonstrated that the overall incidence of complications from laparoscopic-assisted surgery was significantly lower than that from open surgery in colorectal cancer patients with VO 24. Additionally, a previous observational study also showed that hand-assisted laparoscopic distal gastrectomy had obvious superiority over open distal gastrectomy in reducing estimated blood loss, wound length, number of analgesic injections, time to the first flatus, and postoperative hospital stay in obese patients 25. Another interesting finding was that all the examined complications were lower in the laparoscopic group than in the open group; however, only the difference in the incidence of surgical complications was statistically significant, which may be owing to the small sample size. Previous studies 26, 27 have indicated that the duration of the operation and the volume of blood loss are greatly associated with morbidity after gastrectomy. Owing to the lack of tactile sensation, a narrow operating field, a complicated vascular structure in the splenic hilum, and the advanced techniques of systemic lymph node dissections, LG is a time-consuming procedure. However, considering that VO significantly increases insulin resistance, decreases oxygen tension within surgical wounds, impairs tissue penetration of perioperative antibiotics, and increases operative blood loss, LG, which has the advantage of less trauma, smaller wounds, and reduced blood loss 28, still has obvious superiority over OG in patients with VO.

Another major concern in performing LG is the long-term survival of patients with GC. As the oncological outcomes of LG are comparable to those associated with OG, studies have revealed that LG could yield similar oncologic outcomes to OG in treating GC 14, 29, 30. However, the relationship between LG and OG in terms of the long-term efficacy in patients with VO has not yet been reported. Interestingly, our results first demonstrated that OS was significantly longer in patients with VO who underwent LG, and laparoscopic surgery was an independent protective factor for OS. It is worth mentioning that LG was not significantly related to OS in unmatched comparison. That was probably because the effect of LG was masked by the difference of these long-term survival-related factors such as tumor staging, age, and nutrition score. However, in the matched comparison, where there were no other differences between the two groups, LG turned out to be significantly related to the OS. This was probably because compared with open surgery, LG has a better surgical vision and a larger cleaning scope for these visceral obesity patients. On the other hand, although these patients were well matched in this study, patients seemed to be younger and lower TNM staging, which also contributed to the active role of LG for favorable long-term outcomes in GC patients with VO. In addition, our findings suggest that the TNM stage and NRS score were also independent prognostic factors for OS, which is similar to the traditional knowledge on the prognostic factors for survival of patients with GC.

To the best of our knowledge, the present study is the first to evaluate the short- and long-term outcomes of LG in GC patients with VO using a PSM analysis. However, we recognize that our study has several limitations. First and most important, this is not a randomized controlled trial, and inherent selection biases can be adjusted but cannot entirely be eliminated by using PSM. Second, this was not a multiple-center study; therefore, our results may not be directly applicable to other populations. Third, a part of the patients went to other medical centers for further treatment, and a few others did not undergo regular out-patient review. Therefore, we could only obtain the time of patient's death by phone and have no information about the recurrence time. As a result, the analysis of disease-free survival (DFS) and progression-free survival (PFS) was only partially complete. Finally, the small sample size after matching may limit the broader applicability of our findings, and a large-sample study is still necessary.

In conclusion, compared with OG, LG can significantly reduce the incidence of postoperative complications in patients with VO, promote postoperative recovery, and provide better long-term efficacy. Therefore, laparoscopic surgery can be strongly recommended surgical for GC patients with VO.

## Figures and Tables

**Figure 2:**
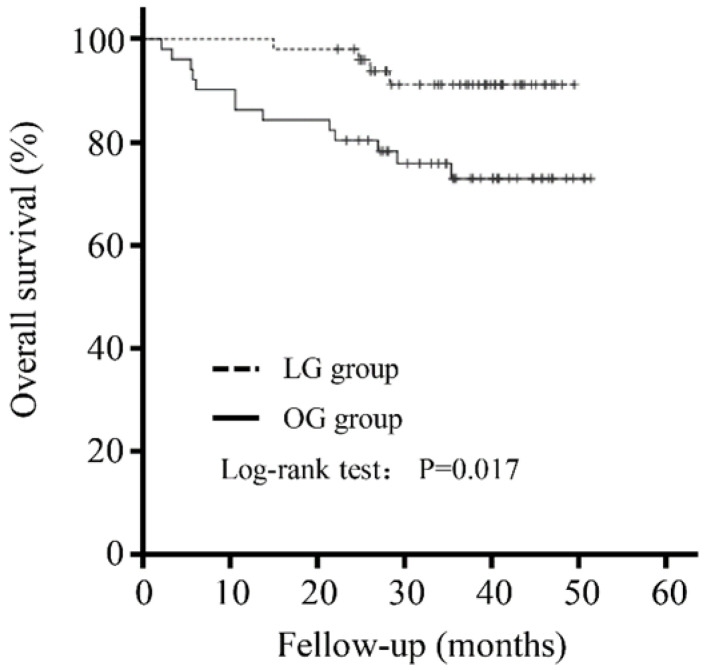
Five-year overall survival curve calculated using the Kaplan-Meier method comparing the LG and OG groups.

**Table 1:** Patient baseline characteristics

Factors	Unmatched comparison			Unmatched comparison		
	Laparoscopic (n=57)	Open (n=188)	*P*	Laparoscopic (n=51)	Open (n=51)	*P*
**Gender**			0.652			0.799
Male	45 (78.95%)	143 (76.06%)		42 (82.35%)	41 (80.39%)	
Female	12 (21.05%)	45 (23.94%)		9 (17.65%)	10 (19.61%)	
**Age (y)**			0.003*			0.074
≤65	35 (61.40%)	74 (39.36%)		32 (62.75%)	23 (45.10%)	
>65	22 (38.60%)	114 (60.64%)		19 (37.25%)	28 (54.90%)	
**BMI (kg/m^2^)**			0.434			0.427
≤18.5	0 (0%)	3 (1.60%)		0 (0%)	0 (0%)	
18.5-24	27 (47.37%)	84 (44.68%)		26 (50.98%)	22 (43.14%)	
>24	30 (52.63%)	101 (53.72%)		25 (49.02%)	29 (56.86%)	
**NRS 2002 score**			0.039*			0.822
1-2	45 (78.95%)	120 (63.83%)		40 (78.43%)	40 (78.43%)	
3-4	11 (19.30%)	52 (27.66%)		10 (19.61%)	9 (17.65%)	
5-6	1 (1.75%)	16 (8.51%)		1 (1.96%)	2 (3.92%)	
**ASA grade**			0.013*			1.000
1-2	52 (91.23%)	143 (76.06%)		46 (90.20%)	47 (92.16%)	
3-4	5 (8.77%)	45 (23.94%)		5 (9.80%)	4 (7.84%)	
**Hypertension**			0.856			1.000
Yes	21 (36.84%)	68 (36.17%)		21 (41.18%)	21 (41.18%)	
No	36 (63.1619.30%)	120 (63.83%)		30 (58.82%)	30 (58.82%)	
**Diabetes mellitus**			0.880			1.000
Yes	11 (19.30%)	38 (20.21%)		9 (17.65%)	9 (17.65%)	
No	46 (80.70%)	150 (79.79%)		42 (82.35%)	42 (82.35%)	
**Previous abdominal surgery**			0.361			0.433
Yes	5 (8.77%)	25 (13.30%)		5 (9.80%)	2 (3.92%)	
No	52 (91.23%)	163 (86.70%)		46 (90.20%)	49 (96.08)	
**Tumor location**			0.480			
Cardia	8 (14.04%)	30 (15.96%)		5 (9.80%)	6 (11.76%)	0.688
Body	15 (26.32%)	33 (17.55%)		13 (25.49%)	13 (25.49%)	
Antrum	33 (57.89%)	118 (62.77%)		32 (62.75%)	32 (62.75%)	
Total	1 (1.75%)	7 (3.72%)		1 (1.96%)	0 (0%)	
**Differentiated degree**			0.952			0.573
Differentiated	44 (77.19%)	142 (75.53%)		39 (76.47%)	35 (68.63%)	
Undifferentiated	5 (8.77%)	19 (10.11%)		5 (9.80%)	5 (9.80%)	
Signet ring carcinoma	8 (14.04%)	27 (14.36%)		7 (13.73%)	11 (21.57%)	
**Pathological type**			0.848			1.000
Ulcerative type	53 (92.98%)	172 (91.49%)		47 (92.16%)	46 (90.20%)	
Non-ulcerative type	4 (7.02%)	16 (8.51%)		4 (7.84%)	5 (9.80%)	
**T stage**			0.028			0.349
I	22 (38.60%)	39 (20.74%)		23 (45.11%)	16 (31.37%)	
II	9 (15.79%)	29 (15.53%)		6 (11.76%)	12 (23.53%)	
III	6 (10.53%)	38 (20.21%)		6 (11.76%)	6 (11.76%)	
IV	19 (33.33%)	82 (43.62%)		16 (31.37%)	17 (33.34%)	
**Lymphatic metastasis number**			0.043			0.204
0	35 (61.40%)	82 (43.62%)		31 (60.78%)	27 (52.94%)	
1-2	10 (17.54%)	35 (18.61%)		9 (17.65%)	10 (19.61%)	
3-6	9 (15.79%)	38 (20.21%)		9 (17.65%)	8 (15.69%)	
7-16	3 (5.3%)	25 (13.30%)		2 (3.92%)	2 (3.92%)	
>16	0 (0%)	7 (3.72%)		0 (0%)	4 (7.84%)	
**TNM stage**			0.006			0.924
I	29 (50.88%)	53 (28.19%)		25 (49.02%)	23 (45.10%)	
II	11 (19.30%)	52 (27.66%)		11 (21.57%)	12 (23.53%)	
III	17 (29.82%)	83 (44.15%)		15 (29.41%)	16 (31.37%)	
**Combined organ resection**			0.387			1.000
Yes	2 (3.51%)	15 (7.98%)		2 (3.92%)	1 (1.96%)	
No	55 (96.49%)	173 (92.02%)		49 (96.08%)	50 (98.04%)	
**Postoperative chemotherapy**			0.007			0.842
Yes	30 (52.63%)	135 (71.81%)		29 (56.86%)	28 (54.90%)	
No	27 (47.37%)	53 (28.19%)		22 (43.14%)	23 (45.10%)	

BMI; body mass index, ASA; American Society of Anesthesiologists, NRS 2002; nutritional risk screening 2002, TNM tumor-node-metastasis; The values represent the number of patients, and values in parentheses represent percentages; *Represents *P* < 0.05, which was considered to be statistically significant.

**Table 2:** Surgical outcomes before and after matching

Factors	Unmatched comparison			Matched comparison		
	LG (n=57)	OG (n=188)	*P*	LG (n=51)	OG (n=51)	*P*
Total complications^a^	7 (12.28%)	74 (39.36%)	<0.001*	6 (11.76%)	22 (43.14%)	<0.001*
**Clavien-Dindo grade**						
Grade I	2 (3.51%)	8 (4.26%)	1.000	1 (1.96%)	4 (7.84%)	0.359
Grade II	4 (7.02%)	44 (23.40%)	0.006*	4 (7.84%)	14 (27.45%)	0.009*
Grade III	1 (1.75%)	14 (7.45%)	0.209	1 (1.96%)	2 (3.92%)	1.000
Grade IV	0 (0.00%)	8 (4.26%)	0.247	0 (0.00%)	2 (3.92%)	0.475
Severe complications^b^	1 (1.75%)	22 (11.71%)	0.024	1 (1.96%)	4 (7.84%)	0.359
**Detail of complications**						
Surgical complications	3 (5.26%)	47 (25.00%)	0.001*	3 (5.88%)	15 (29.41%)	0.002*
Gastrointestinal dysfunction	0 (0.00%)	7 (3.72%)	0.306	0 (0.00%)	5 (9.80%)	0.067
Intestinal obstruction	0 (0.00%)	5 (2.66%)	0.478	0 (0.00%)	2 (3.92%)	0.475
Anastomotic leakage	0 (0.00%)	5 (2.66%)	0.478	0 (0.00%)	2 (3.92%)	0.475
Severe wound infection	0 (0.00%)	4 (2.13%)	0.576	0 (0.00%)	1 (1.96%)	1.000
Intra-abdominal infection	2 (3.51%)	19 (10.11%)	0.198	2 (3.92%)	3 (5.88%)	1.000
Intra-abdominal Bleeding	1 (1.75%)	7 (3.72%)	0.759	1 (1.96%)	2 (3.92%)	1.000
Medical complications	4 (7.02%)	27 (14.36%)	0.144	3 (5.88%)	7 (13.73%)	0.183
Pleural and peritoneal effusion	4 (7.02%)	11 (5.85%)	0.995	3 (5.88%)	4 (7.84%)	1.000
Pulmonary complications	0 (0.00%)	8 (4.26%)	0.247	0 (0.00%)	1 (1.96%)	1.000
Venous thrombosis	0 (0.00%)	8 (4.26%)	0.247	0 (0.00%)	2 (3.92%)	0.475
30-day mortality	0 (0.00%)	3 (1.60%)	1.000	0 (0.00%)	0 (0.00%)	-
Operative time, (X ± SD), min	227.87±47.47	200.46±49.42	<0.001*	226.29±48.74	191.25±52.23	0.001*
Postoperative hospital stays, (X ± SD), days	11.67±3.87	17.12±9.25	<0.001*	11.73±4.03	17.45±9.28	<0.001*
Hospitalization costs, (X ± SD), yuan	62574.56±18724.56	66928.08±35050.36	0.370	62763.13±19705.45	63020.28±28839.08	0.958

Values are shown as n (%) unless otherwise indicated. ^a^Postoperative complications in this study were defined as any adverse event corresponding to Clavien-Dindo classification grade, occurring within 30 days after surgery. If a patient had more than one type of complication, the complication with the highest grade was used for the analysis. ^b^Clavien-Dindo grade ≥ III. *P<0.05, statistically significant.

**Table 3:** Univariate and multivariate logistic analysis of factors associated with total postoperative complications

Factors	Unmatched comparison				Matched comparison			
	Univariate analysis		Multivariate analysis		Univariate analysis		Multivariate analysis	
	OR (95% CI)	*P*	OR (95% CI)	*P*	OR (95% CI)	*P*	OR (95% CI)	*P*
**Gender**								
Male	Ref				Ref			
Female	0.984 (0.525-1.846)	0.118			2.291 (0.809-6.484)	0.118		
**Age (y)**								
≤65	Ref				Ref			
>65	1.847 (1.065-3.204)	0.029			1.514 (0.632-3.628)	0.352		
**BMI (kg/m^2^)**								
18.5-24	Ref	0.365			Ref	0.212		
>24	0.780 (0.456-1.335)				0.571 (0.237-1.376)			
**NRS 2002 score**								
1-2	Ref				Ref			
3-4	1.234 (0.667-2.283)	0.503			1.297 (0.437-3.849)	0.640		
5-6	2.587 (0.944-7.094)	0.065			1.405 (0.121-16.305)	0.786		
**ASA grade**								
1-2	Ref				Ref			
3-4	1.179 (0.615-2.259)	0.621			0.736 (0.143-3.778)	0.714		
**Hypertension**								
No	Ref				Ref			
Yes	0.967 (0.555-1.683)	0.905			0.898 (0.369-2.181)	0.811		
**Diabetes mellitus**								
No	Ref				Ref			
Yes	1.369 (0.715-2.619)	0.343			0.714 (0.213-2.390)	0.585		
**Previous abdominal surgery**								
No	Ref				Ref			
Yes	1.014 (0.4518-2.281)	0.973			0.420 (0.048-3.652)	0.432		
**Tumor location**								
Cardia	Ref				Ref			
Body	0.459 (0.186-1.131)	0.091			0.286 (0.061-1.328)	0.110		
Antrum	0.593 (0.288-1.225)	0.158			0.470 (0.127-1.733)	0.257		
Total	0.412 (0.073-2.307)	0.313				1.000		
**Differentiated degree**								
Differentiated	Ref				Ref			
Undifferentiated	0.844 (0.332-2.143)	0.721			0.300 (0.036-2.521)	0.208		
Signet ring carcinoma	1.211 (0.571-2.566)	0.617			1.718 (0.585-5.047)	0.325		
**Pathological type**								
Ulcerative type	Ref				Ref			
Non-ulcerative type	1.414 (0.600-3.331)	0.428			0.736 (0.143-3.778)	0.714		
**TNM stage**								
I	Ref				Ref			
II	1.487 (0.741-2.984)	0.264			1.929 (0.667-5.578)	0.226		
III	1.190 (0.632-2.240)	0.589			0.875 (0.301-2.540)	0.803		
**Combined organ resection**								
No	Ref				Ref			
Yes	1.113 (0.396-3.124)	0.839			1.333 (0.116-15.311)	0.817		
**Laparoscopic gastrectomy**								
No	Ref		Ref		Ref		Ref	
Yes	0.216 (0.093-0.501)	<0.001	0.215 (0.087-0.530)	0.001	0.176 (0.064-0.484)	0.001	0.141 (0.042-0.472)	0.001

BMI body mass index; ASA American Society of Anesthesiologists, NRS 2002 nutritional risk screening 2002, TNM tumor-node-metastasis. *Statistically significant (P < 0.05).

**Table 4:** Univariate and multivariate Cox regression analysis of factors associated with overall survival

Factors	Unmatched comparison				Matched comparison			
	Univariate analysis		Univariate analysis		Univariate analysis		Univariate analysis	
	HR (95% CI)	*P*	HR (95% CI)	*P*	HR (95% CI)	*P*	HR (95% CI)	*P*
**Gender**								
Male	Ref				Ref			
Female	0.908 (0.318-2.773)	0.908			0.923 (0.265-3.214)	0.900		
**Age (y)**								
≤65	Ref				Ref			
>65	1.083 (0.483-2.420)	0.846			2.320 (0.857-6.276)	0.098		
**BMI (kg/m^2^)**								
18.5-24	Ref				Ref			
>24	0.710 (0.328-1.538)	0.385			2.222 (0.783-6.311)	0.134		
**NRS 2002 score**								
1-2	Ref		Ref		Ref		Ref	
3-4	1.687 (0.719-3.955)	0.229	1.142 (0.470-2.771)	0.770	1.698 (0.540-5.341)	0.366	2.588 (0.579-11.570)	0.213
5-6	3.106 (1.061-9.091)	0.039*	1.647 (0.533-5.083)	0.386	6.180 (1.301-28.055)	0.018*	12.968 (1.780-94.495)	0.011*
**ASA grade**								
1-2	Ref				Ref			
3-4	1.217 (0.497-2.983)	0.667			2.085 (0.599-7.257)	0.248		
**Hypertension**								
No	Ref				Ref			
Yes	0.507 (0.229-1.123)	0.094			0.743 (0.275-2.010)	0.559		
**Diabetes mellitus**								
No	Ref				Ref			
Yes	0.418 (0.143-1.226)	0.112			0.959 (0.275-3.342)	0.948		
**Previous abdominal surgery**								
No	Ref				Ref			
Yes	1.643 (0.612-4.408)	0.324			0.829 (0.110-6.256)	0.856		
**Tumor location**								
Cardia	Ref				Ref			
Body	1.102 (0.274-4.435)	0.891			1.071 (0.208-5.521)	0.935		
Antrum	1.182 (0.391-3.569)	0.767			0.717 (0.155-3.323)	0.671		
Total	3.909 (0.699-21.861)	0.121			6.520 (0.582-72.979)	0.128		
**Differentiated degree**								
Differentiated	Ref				Ref			
Undifferentiated	1.214 (0.400-3.680)	0.732			1.310 (0.293-5.864)	0.724		
Signet ring carcinoma	1.257 (0.460-3.433)	0.655			1.033 (0.291-3.601)	0.960		
**Pathological type**								
Ulcerative type	Ref				Ref			
Non-ulcerative type	0.865 (0.256-2.925)	0.816			1.732 (0.395-7.596)	0.467		
**TNM stage**								
I	Ref		Ref		Ref		Ref	
II	9.633 (1.154-80.384)	0.036*	9.815 (1.174-82.049)	0.035*	4.758 (0.871-25.995)	0.072	5.686 (0.737-43.861)	0.095
III	14.208 (1.898-106.340)	0.010*	10.735 (1.386-83.148)	0.023*	10.492 (2.321-47.434)	0.002*	17.492 (2.445-125.157)	0.004*
**Combined organ resection**								
No	Ref				Ref			
Yes	1.630 (0.381-6.969)	0.510			2.425 (0.321-18.315)	0.391		
**Laparoscopic gastrectomy**								
No	Ref		Ref		Ref		Ref	
Yes	0.135 (0.018-1.002)	0.050*	0.178 (0.024-1.343)	0.094	0.280 (0.091-0.859)	0.026*	0.264 (0.077-0.905)	0.034*

BMI body mass index; ASA American Society of Anesthesiologists, NRS 2002 nutritional risk screening 2002, TNM tumor-node-metastasis. *Statistically significant (P < 0.05).
